# MetaDAVis: An R shiny application for metagenomic data analysis and visualization

**DOI:** 10.1371/journal.pone.0319949

**Published:** 2025-04-07

**Authors:** Sankarasubramanian Jagadesan, Chittibabu Guda

**Affiliations:** 1 Department of Genetics, Cell Biology and Anatomy, University of Nebraska Medical Center, Omaha, Nebraska, United States of America; 2 Center for Biomedical Informatics Research and Innovation, University of Nebraska Medical Center, Omaha, Nebraska, United States of America; University of Helsinki: Helsingin Yliopisto, FINLAND

## Abstract

The human microbiome exerts tremendous influence on maintaining a balance between human health and disease. High-throughput sequencing has enabled the study of microbial communities at an unprecedented resolution. Generation of massive amounts of sequencing data has also presented novel challenges to analyzing and visualizing data to make biologically relevant interpretations. We have developed an interactive Metagenome Data Analysis and Visualization (MetaDAVis) tool for 16S rRNA as well as the whole genome sequencing data analysis and visualization to address these challenges using an R Shiny application. MetaDAVis can perform six different types of analyses that include: i) Taxonomic abundance distribution; ii) Alpha and beta diversity analyses; iii) Dimension reduction tasks using PCA, t-SNE, and UMAP; iv) Correlation analysis using taxa- or sample-based data; v) Heatmap generation; and vi) Differential abundance analysis. MetaDAVis creates interactive and dynamic figures and tables from multiple methods enabling users to easily understand their data using different variables. Our program is user-friendly and easily customizable allowing those without any programming background to perform comprehensive data analyses using a standalone or web-based interface.

## Introduction

It is estimated that human bodies are inhabitated by an average of 500 - 1000 different microbial species [1,2]. The complex role of human microbiome in shaping human health and disease has been extensively investigated in recent years due to the advancement of metagenome sequencing technologies [3–5]. Generation of massive volumes of sequencing data poses new challenges for the data analytics and interpretation of results in the microbiome research. Likewise, advanced analytical and visualization tools in this research arena can improve our ability to understand the roles of microbes in diverse environments and how they interact with each other and their human hosts. Thorough analysis of microbiome data consists of two essential components: the upstream community profiling that include alpha and beta diversity analysis, taxonomic profiling and abundance estimation and the downstream characterization of microbial communities such as differential abundance estimation and functional and metabolic profiling.

In recent years, several data analysis and visualization methods have been developed for microbiome data analysis [6–8]. For 16S rRNA, raw sequence reads were initially processed and clustered into operational taxonomical units (OTUs) using a variety of reputed tools, such as MOTHUR [9] or QIIME [10] and QIIME2 [11]. Similarly, the whole genome sequencing reads were processed using DIAMOND+MEGAN [12], which aligns all reads against a protein reference database. Similarly, tools such as Vegan [13], MicrobiomeAnalyst [14], MicobiomeR [15], Metavizr [16], Microbiome helper [17], Phyloseq [18], Animalcules [19], WisDOM [20] were developed for data analysis and visualization with each tool having varying capabilities and limitations. A comparative analysis of different tasks performed by the popular metagenomics tools is provided in Table 1.

**Table 1:** Comparison of MetaDAVis and other popular microbiome analysis tools.

Functionality Module	Vegan	Mothur	MicrobiomeAnalyst	MicrobiomeR	Metavizr	Microbiome helper	Qiime2	Phyloseq	Animalcules	wiSDOM	MetaDAVis
Support for 16S rRNA data	✔	✔	✔	✔	✔	✔	✔	✔	✔	✔	✔
Support for WGS data	✔			✔				✔	✔		✔
Data summary and distribution plot										✔	✔
Diversity analysis	✔	✔	✔	✔	✔	✔	✔	✔	✔	✔	✔
Dimension reduction		✔	✔	✔	✔	✔	✔	✔	✔		✔
Differential abundance analysis (two groups)		✔	✔	✔	✔	✔	✔	✔	✔	✔	✔
Differential abundance analysis (multiple groups)											✔
Heatmap											✔
Correlation analysis											✔
command-line	✔	✔		✔		✔	✔	✔	✔	✔	✔
Interactive visualization (Plots and tables GUI)			✔		✔		✔		✔	✔	✔
Web interface			✔					✔		✔	✔
Language/platform	R	R	R	R	R	R/Perl/Python	Python	R	R	R	R

Current metagenomics tools mainly support taxonomic profiling and abundance estimation, alpha and beta diversity analysis, dimension reduction visualization, and differential abundance estimation. Similarly, many independent statistical and machine-learning approaches have been developed to perform the above tasks which were not well integrated with these methods limiting the options for users to test and visualize the output from different tools. For instance, eventhough QIIME2 and Mothur provide excellent analytical and visualization tools, they do not support the whole genome sequencing (WGS) data analysis. Microbiome helper has a collection of scripts in multiple programming languages to facilitate interaction and interoperability among multiple tools but offers limited interactive visualization capabilities. On the other hand, STAMP [21] enables statistical analysis of taxonomic and functional profiles with various visualization tools, but it lacks abundance distribution and diversity index analyses. Some of the R-based metagenomics packages such as microbiomeR package provides only command-line workflows. Metavizr offers a graphical-user interface (GUI) with limited metagenomic visualizations. Similarly, R Shiny-based applications such as Phyloseq has useful tools for annotation, visualization, and diversity analysis but does not provide abundance analysis. More recent tools such as Animalcules, offers good interactive features in command-line and GUI modes for alpha/beta diversity and differential abundance analysis between two conditional groups but not for the multiple group comparisions. However, this program lacks a web interface. Lastly, wiSDOM, an R shiny standalone and web-based application provides many diversity profiling and statistical analysis functions but only works with the 16S rRNA data (Table 1). Moreover, most of the existing tools also require programming expertise and significant effort from the user to install and configure different programming languages such as R, Matlab, and Python on local servers. To address most of these issues, here we present an interactive Metagenome Data Analysis and Visualization (MetaDAVis) tool using an R-based Shiny application and web interface. The rich set of features offered by MetaDAVis are presented in Table 1 in comparison to the existing methods. There are six functional modules offered by our tool, where each module can perform a subset of tasks based on the chosen option using multiple methods. The novely of MetaDAVis lies in its design that enables it to function interactively by taking the user’s choice of methods and variables as input and provide publication-quality plots and result tables that can be downloaded in different formats.

## Design and implementation

Multiple R packages listed in S1 Table were used to create and implement MetaDAVis, which can be installed through Github. It requires R package version 4.4.2 or higher and Shiny package version 1.10.0 or higher. After loading the dependent libraries in R, users can launch the R Shiny GUI on a desktop using shiny::runGitHub(“MetaDAVis”, “gudalab”), or access on the web at the URL: https://www.gudalab-rtools.net/MetaDAVis (S1 Fig). Example datasets used for the development work include 16S rRNA (NCBI SRA: SRP128892) [22] and whole genome sequencing reads (NCBI SRA: SRP108707) [23] from inflammatory bowel disease, which were processed using Qiime2, Diamond and MEGAN in our previous studies [24].

### Input file formats

Our application accepts files in.txt,.tsv, or.csv format. Users can directly upload Level 7 Qiime2 results generated using Greengenes or Silva. Additionally, it supports MEGAN data from whole metagenome sequences (ensure to remove the metadata column if included in the level7.csv file from Qiime2). For Qiime2 input files, the first column serves as an index containing sample IDs, while the second to Nth columns represent taxonomy (S2A Fig). For MEGAN input files, the first seven columns (Level_1 to Level_7) are followed by sample names (S2B Fig). If users wish to upload their files, the first seven columns should contain Kingdom, Phylum, Class, Order, Family, Genus, and Species, followed by sample names (S2C Fig). Metadata files must have two columns. The first column should list sample IDs that match those in the count data input, while the second column, Condition, indicates a user-defined categorical variable, such as “case” and “control” (for two or more groups) (S2D Fig). Users can also refer to our example count data and metadata files available on the tool’s upload page (S3 Fig) or example datasets from our GitHub repository (https://github.com/GudaLab/MetaDAVis).

### Guidelines

Once the Input files are uploaded, each of the six modues in MetaDAVis can be used independently in any order. This tool was tested in Linux (RedHat and Ubuntu) and Windows 10 and 11. A user’s manual can be accessed at https://www.gudalab-rtools.net/MetaDAVis/manual/MetaDAVis_manual.pdf to aid in the installation and usage of the application. Summary tables were developed using the DataTables (DT) package to display results in up to 100 rows, while the entire tables can be downloaded as.csv files. Similarly, graphical plots were downloaded in multiple formats using the downloadHandler function from shiny packages. We implemented custom color options using the *RColorBrewer* package, allowing users to download the figures in the same color format coding format in all the modules except for correlation analysis and MaAsLin3 results. We also provided example data (‘Example Data (To test our tool)’ under the ‘Select Input Format’ section. This feature helps demonstrate our tool’s performance, offering reassurance to users

## Results

**Fig 1:**
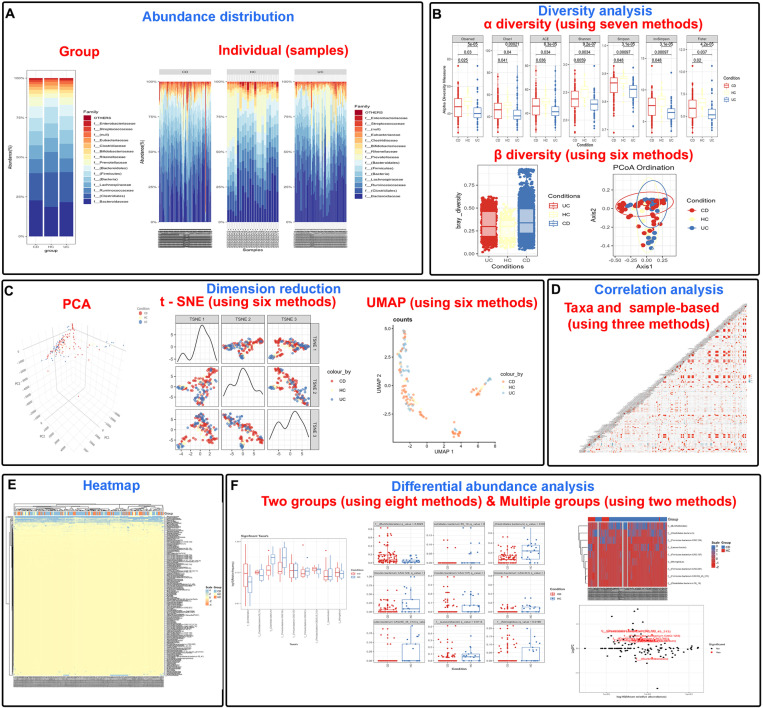
Workflow and example output of MetaDAVis. (a) Group and sample-based abundance stacked plot, (b) Alpha diversity and beta diversity box plot, (c) 3D PCA, t-SNE and UMAP orientation plots, (d) taxa and sample-based correlation plot, (e) heatmap of the abundance values, (f) differential abundance analysis generates boxplots for grouped and individual significant taxa, volcano plot and heatmap; two groups implemented with Wilcoxon Rank Sum, t-test, methagenomeSeq, DESeq2, Limma-Voom, edgeR, lefser, MaAsLin3; Multiple groups were implemented with Kruskal-Wallis test and ANOVA.

MetaDAVis is a versatile program that accepts the output of several primary data analysis tools such as MEGAN or Qiime2 as input, performs interactive downstream analyses, and generates a variety of visually appealing plots in different formats that can be directly used for presentations and publications. We designed MetaDAVis to include six functional modules that cover the commonly used tasks in metagenomic data analysis. These include 1) Taxonomic distribution, 2) Taxonomic diversity, 3) Dimension reduction, 4) Correlation analysis, 5) Heatmap generation, and 6) Differential abundance analysis (between two or more groups). Each module performs a distinct task in the workflow, where users have the ability to select various thresholds and algorithmic and visualization parameters to generate custom plots. Fig 1 illustrates the outputs from the tasks performed by MetaDAVis. All R packages with corresponding references, github links, and the task performed by each module are summarized in S1 Table. Publishing quality results from MetaDAVis analysis can be downloaded in seven different formats such as JPG, TIFF, PDF, SVG, BMP, EPS, and PS.

### Data summary and distribution analysis

MetaDAVis accepts the output formats of Qiime2 (generated using Greengenes or Silva) or MEGAN (.csv &.tsv) as input to generate relative abundance and taxonomic distribution plots as per the user selected options (Fig 1A). Relative abundance plots can be generated at seven hierarchical taxonomic levels (Kingdom, Phylum, Class, Order, Family, Genus, and Species) and results can be visualized in multiple plots and tables. For example, a distribution box plot for individual samples and their comparison groups was shown in S4A–S4C Fig.

### Diversity analysis

Alpha diversity is calculated using read count or relative abundance data within a sample and compared between groups. We have implemented seven different methods including *Observed, Chao1, ACE, Shannon, Simpson, Inverse Simpson, and Fisher* from the phyloseq package [18] for α-diversity calculation and the results can be visualized as box or violin plots (Fig 1B) with their summary table (S5A–S5D Fig). Users can also perform the Wilcoxon test to display the statistical significance (p-value) using the *microbiomeutilities* package [25].

Beta diversity was calculated using phyloseq and vegan [13] packages (Fig 1B). We have incorporated the adonis2 function and defined the parameters such as diversity methods, number of permutations, square root of dissimilarities. Users can choose any one of the methods (bray-curtis, jaccard, manhattan, euclidean, canberra, kulczynski, gower, altGower, morisita, horn, clark, mountford, raup, binomial, chao, cao, mahalanobis, chisq, chord, hellinger, aitchison, and robust.aitchison) for beta diversity calculation using the integrated distance matrices. In addition, users can select any machine learning algorithm including PCoA, NMDS, DCA, RDA, and MDS for assessing the between-sample microbial diversity (S5E–S5I Fig).

### Dimension reduction

A critical step in any data analysis is visualizing and summarizing highly variable data in a lower-dimensional space. We implement two and three commonly used dimensionality reduction techniques (Fig 1C) including principal components analysis (PCA) in 2D and 3D with coord_equal(ratio =  1) to get the consistent scale [26], t-distributed stochastic neighbor embedding (t-SNE) [27], and uniform manifold approximation and projection (UMAP) [28]. PCA is a linear dimensionality reduction method where the first three axes explain maximum amount of variation. In contrast, t-SNE and UMAP are non-linear methods for mapping data to a lower-dimensional embedding. We have incorporated six methods from the *scater* package [29] to plot the t-SNE and UMAP: *counts, rclr, hellinger, pa, rank,* and *relabundance* to plot the dimension reduction. The plotted dimension reduction values were provided in separate tables in (S6A–S6L Fig).

### Correlation analysis

We implemented both taxon-based and sample-based correlations using *GGally*, which is an extension to [30] using *ggcorr* function to call Pearson, Kendall, and Spearman methods (Fig 1D). Users can check the correlation for each condition separately or select multiple options together using the dropdown menu. Similarly, sample-based correlations can be calculated separately for each group of samples under specific conditions or combined across conditions. Correlation plots and summary matrices can be generated by the user with their method of choice (S7A–S7C Fig).

### Generating heatmaps

To visualize the relative abundance diversity among the samples, we have implemented a heatmap using *ComplexHeatmap* [31] and *scales* [32], with multiple options to display or hide the row and column names and cladograms, clustering methods for rows and columns using options such as single, complete, average (UPGMA), mcquitty (WPGMA), median (WPGMC), and centroid (UPGMC), normalization methods, such as scale, minmax, log, row normalization, column normalization, and none (Fig 1E; S8A and S8B Fig).

### Differential abundance analysis

For pair-wise comparison, the generalized linear model-based methods, including *DESeq2* [33] and *edgeR* [34], *Two-sample t-test*, *Wilcoxon Rank Sum test, metagenomeSeq* [35], *limma-voom* [36]*,* Linear Discriminant Analysis Effect Size (LEfSe) *lefser* [37] and (Microbiome Multivariable Association with Linear Models) *MaAsLin3* [38] were used to identify the taxa with different abundances in two different groups. We converted the raw count value to relative frequency using the formula (**Relative Frequency = ** (**Subgroup frequency/ Total frequency**) * 100)) for the Wilcoxon Rank Sum test (wilcox.test) and t-test (t.test) statistical analyses. However, *metagenomeSeq*, *DESeq2*, *Limma-Voom*, *edgeR lefser* and *MaAsLin3* have in-built algorithms to find statistically significant biomarkers. For multiple testing, biomarker candidates can be filtered using user-specified p-value or false discovery rate (FDR, q-value) from the Benjamini-Hochberg procedure [39]. Users have the flexibility to adjust the FDR or p-value based on their needs (default is <  0.05). Results can be downloaded either as the grouped or individual box plot for each taxon, or as volcano plots or heatmaps of significantly identified taxa (Fig 1F; S9A–S9F Fig) with summary tables (S2 Table). MaAsLin3 generates multiple tables and figures, and we provide these result files in a compressed zip format for ease of access.

For analyses involving multiple-group comparisons such as control, case 1 and case 2, we implemented the Kruskal-Wallis (*kruskal.test*) and ANOVA (Analysis of variance) to identify differentially abundant taxonomic markers. In addition, post-hoc test that calculates p-values for pairwise comparisons among the members of the group was implemented using *dunn.test* package in the Kruskal-Wallis test. Likewise, *TukeyHSD* was used under ANOVA testing. We have applied the Benjamini-Hochberg FDR or p-value and the post hoc test. These results can be downloaded similar to those from the two-group comparisons.

MetaDAVis provides a graphical user interface through R/Shiny, which can be used even by those without prior programming knowledge. The tools such as vegan, Mothur, MicrobiomeR, Microbiomehelper, and Qiime2 are only command-line interfaces, which limits their usage without prior programming experience. Furthermore, several tools (as presented in Table 1) impose the burden of importing/exporting additional packages, which also requires programming skills. MetaDAVis can be installed locally for standalone use or accessed via an user-friendly web interface to analyze both 16S rRNA and WGS data (Table 1). Hence, it offers more flexibility for use by both seasoned programmers and non-programmers. MataDAVis application is embedded with rich sets of options in each module to choose a variety of methods and perform highly customizable analyses for microbiome sequencing data. For example, it allows users to analyze data at seven different multiple taxonomic levels, provides multiple options for data normalization and distribution analysis, facilitates visualization of data using PCA, t-SNE, UMAP with multiple methods within each approach, and similarly, offers multiple methods to carry out differential abundance analysis, and supports differential analysis and visualization for pairwise and group comparisons. Each of the six modules in MetaDAVis can be used in no particular order using outputs from other methods as inputs, which adds a lot flexibility for users to build and carryout customizable data analysis pipelines. The primary advantage of using MetaDAVis over existing methods is the ease of accessing many independent statistical and machine-learning tools all in one platform, seamlessely, to carryout highly specialized and refined microbiome data analyses using 16S rRNA or WGS datasets. Another advantage is its rich set of visualization tools and graphical outputs. Each module generates publishing quality plots and summary tables, where the images can be downloaded in seven different formats and the tables are downloaded as.CSV files for further use. MetaDAVis is highly flexible for customization of data pipelines and it can be broadly used without any programming background. We believe that MetaDAVis tool is a unique and highly versatile platform that broadly supports microbiome research.

## Supporting information

S1 Table List of R packages used to develop MetaDAVis. (DOCX)

S2 Table Output table for the significant taxa by using various methods. (DOCX)

S1 Fig The MetaDAVis web application. (TIF)

S2 Fig Supported input file formats for MetaDAVis. (A) Qiime 2 output (Level 7), (B) MEGAN output file, (C) user-defined file format, and (D) metadata file applicable to all three formats.
(TIF)

S3 Fig Data upload page with display options and example summary display. Example data were provided for Qiime2, MEGAN output format. If users have a different output format, they should be prepared according to the taxa count file format.
(TIF)

S4 Fig Distribution plots. (A) Choice of distribution plot and output format; (B) Box plot for comparison groups; and (C) Box plots for individual samples.
(TIF)

S5 Fig Diversity analysis. (A) Choice of alpha diversity method from seven different methods such as Observed, Chao1, ACE, Shannon, Simpson, Inverse Simpson, Fisher or All_combined; (B) Violin plot showing the Simpson diversity; (C and D) Shannon diversity plot with corresponding values in a table; (E) Selected choice of beta diversity methods (bray-curtis) with other options;; corresponding (F) bar plot (G) dot plot (H) values in a table and (I) adonis2 function table.
(TIF)

S6 Fig Orientation analysis. (A-C) The choice of PCA-2D and the plot with frames and summary table of sample coordinate positions shown for PC1 and PC2; (D-F) PCA 3-D selection and the 3-D plot and summary table of sample coordinate positions shown for PC1, PC2, and PC3; (G-I) t-SNE with selected options, two-dimension plots with the selected rcl method, and corresponding summary table; (J-L) UMAP with selected options, condition-based and cluster-based (K = 5) UMAP plots with selected rcl method, and corresponding summary table.
(TIF)

S7 Fig Correlation analysis. (A) Input selection for taxa-based correlation analysis using the condition option; (B) Taxa-based correlation plot using Pearson method; and (C) summary table. A similar type of method selection and results were implemented in sample-based correlation analysis.
(TIF)

S8 Fig Heatmap generation. (A) Input selection for heatmap analysis, user can adjust the row and column text size and cladograms; and (B) Heatmap for the selected taxonomy level shows sample names in rows and family names in columns with a cladogram. Scale values represent colors in the heatmap and condition groups.
(TIF)

S9 Fig Differential abundance analysis. (A) Input selection of Wilcoxon Rank Sum test; (B) Grouped box plot, x-axis represents taxa and y-axis represents log10(relative frequency); (C) An individual box plot for each taxon, x-axis represents the condition and y-axis represents relative frequency; (D) Volcano plot, x-axis represents log10(mean relative abundance) and y-axis represents Log2FC; (E) Heatmap for significantly identified taxa; (F) Summary table for the Wilcoxon Rank Sum test. Similar input is needed for the remaining pairwise methods such as, metagenomeSeq, DESeq2, Limma-Voom and edgeR and multiple group comparison Kruskal-Wallis test and ANOVA.
(TIF)

## References

[1] Gilbert JA, Blaser MJ, Caporaso JG, Jansson JK, Lynch SV, Knight R. Current understanding of the human microbiome. Nat Med. 2018;24(4):392–400. doi: 10.1038/nm.4517 29634682 PMC7043356

[2] Lloyd-Price J, Abu-Ali G, Huttenhower C. The healthy human microbiome. Genome Med. 2016;8(1):51. doi: 10.1186/s13073-016-0307-y 27122046 PMC4848870

[3] Agustinho DP, Fu Y, Menon VK, Metcalf GA, Treangen TJ, Sedlazeck FJ. Unveiling microbial diversity: harnessing long-read sequencing technology. Nat Methods. 2024;21(6):954–66. doi: 10.1038/s41592-024-02262-1 38689099 PMC11955098

[4] Lema NK, Gemeda MT, Woldesemayat AA. Recent Advances in Metagenomic Approaches, Applications, and Challenge. Curr Microbiol. 2023;80(11):347. doi: 10.1007/s00284-023-03451-5 37733134

[5] Zhou Y, Liu M, Yang J. Recovering metagenome-assembled genomes from shotgun metagenomic sequencing data: Methods, applications, challenges, and opportunities. Microbiol Res. 2022;260:127023. doi: 10.1016/j.micres.2022.127023 35430490

[6] Arango-Argoty G, Garner E, Pruden A, Heath LS, Vikesland P, Zhang L. DeepARG: a deep learning approach for predicting antibiotic resistance genes from metagenomic data. Microbiome. 2018;6(1):23. doi: 10.1186/s40168-018-0401-z 29391044 PMC5796597

[7] Qu K, Guo F, Liu X, Lin Y, Zou Q. Application of Machine Learning in Microbiology. Front Microbiol. 2019;10:827. doi: 10.3389/fmicb.2019.00827 31057526 PMC6482238

[8] Zhou Y-H, Gallins P. A Review and Tutorial of Machine Learning Methods for Microbiome Host Trait Prediction. Front Genet. 2019;10:579. doi: 10.3389/fgene.2019.00579 31293616 PMC6603228

[9] Schloss PD, Westcott SL, Ryabin T, Hall JR, Hartmann M, Hollister EB, et al. Introducing mothur: open-source, platform-independent, community-supported software for describing and comparing microbial communities. Appl Environ Microbiol. 2009;75(23):7537–41. doi: 10.1128/AEM.01541-09 19801464 PMC2786419

[10] Caporaso JG, Kuczynski J, Stombaugh J, Bittinger K, Bushman FD, Costello EK, et al. QIIME allows analysis of high-throughput community sequencing data. Nat Methods. 2010;7(5):335–6. doi: 10.1038/nmeth.f.303 20383131 PMC3156573

[11] Bolyen E, Rideout JR, Dillon MR, Bokulich NA, Abnet CC, Al-Ghalith GA, et al. Reproducible, interactive, scalable and extensible microbiome data science using QIIME 2. Nat Biotechnol. 2019;37(8):852–7. doi: 10.1038/s41587-019-0209-9 31341288 PMC7015180

[12] Bağcı C, Patz S, Huson DH. DIAMOND+MEGAN: Fast and Easy Taxonomic and Functional Analysis of Short and Long Microbiome Sequences. Curr Protoc. 2021;1(3):e59. doi: 10.1002/cpz1.59 33656283

[13] Dixon P. VEGAN, a package of R functions for community ecology. J Vegetation Science. 2003;14(6):927–30. doi: 10.1111/j.1654-1103.2003.tb02228.x

[14] Dhariwal A, Chong J, Habib S, King IL, Agellon LB, Xia J. MicrobiomeAnalyst: a web-based tool for comprehensive statistical, visual and meta-analysis of microbiome data. Nucleic Acids Res. 2017;45(W1):W180–8. doi: 10.1093/nar/gkx295 28449106 PMC5570177

[15] Lahti L, Shetty S. microbiome R package. 2017. Available from: http://microbiome.github.io

[16] Bravo HC, Chelaru F, Wagner J, Kancherla J, Paulson J. R Interface to the metaviz web app for interactive metagenomics data analysis and visualization. 2017. Available from: https://epiviz.github.io/metaviz/documentation/IntroToMetavizr.html

[17] Comeau AM, Douglas GM, Langille MGI. Microbiome Helper: a Custom and Streamlined Workflow for Microbiome Research. mSystems. 2017;2(1):e00127-16. doi: 10.1128/mSystems.00127-16 28066818 PMC5209531

[18] McMurdie PJ, Holmes S. phyloseq: an R package for reproducible interactive analysis and graphics of microbiome census data. PLoS One. 2013;8(4):e61217. doi: 10.1371/journal.pone.0061217 23630581 PMC3632530

[19] Zhao Y, Federico A, Faits T, Manimaran S, Segrè D, Monti S, et al. animalcules: interactive microbiome analytics and visualization in R. Microbiome. 2021;9(1):76. doi: 10.1186/s40168-021-01013-0 33775256 PMC8006385

[20] Su S-C, Galvin JE, Yang S-F, Chung W-H, Chang L-C. wiSDOM: a visual and statistical analytics for interrogating microbiome. Bioinformatics. 2021;37(17):2795–7. doi: 10.1093/bioinformatics/btab057 33515241 PMC8428577

[21] Parks DH, Tyson GW, Hugenholtz P, Beiko RG. STAMP: statistical analysis of taxonomic and functional profiles. Bioinformatics. 2014;30(21):3123–4. doi: 10.1093/bioinformatics/btu494 25061070 PMC4609014

[22] Pascal V, Pozuelo M, Borruel N, Casellas F, Campos D, Santiago A, et al. A microbial signature for Crohn’s disease. Gut. 2017;66(5):813–22. doi: 10.1136/gutjnl-2016-313235 28179361 PMC5531220

[23] Hall AB, Yassour M, Sauk J, Garner A, Jiang X, Arthur T, et al. A novel Ruminococcus gnavus clade enriched in inflammatory bowel disease patients. Genome Med. 2017;9(1):103. doi: 10.1186/s13073-017-0490-5 29183332 PMC5704459

[24] Sankarasubramanian J, Ahmad R, Avuthu N, Singh AB, Guda C. Gut Microbiota and Metabolic Specificity in Ulcerative Colitis and Crohn’s Disease. Front Med (Lausanne). 2020;7:606298. doi: 10.3389/fmed.2020.606298 33330572 PMC7729129

[25] Sudarshan S, Leo L. microbiomeutilities: microbiomeutilities: Utilities for Microbiome Analytics. 2022. Available from: https://microsud.github.io/microbiomeutilities/

[26] Pearson K. LIII. On lines and planes of closest fit to systems of points in space. The London, Edinburgh, and Dublin Philosophical Magazine and Journal of Science. 1901;2(11):559–72. doi: 10.1080/14786440109462720

[27] van der Maaten L, Hinton G. Visualizing data using t-SNE. J Mach Learn Res. 2008;9:2579–605.

[28] McInnes L, Healy J, Saul N, Großberger L. UMAP: Uniform Manifold Approximation and Projection. JOSS. 2018;3(29):861. doi: 10.21105/joss.00861

[29] McCarthy DJ, Campbell KR, Lun ATL, Wills QF. Scater: pre-processing, quality control, normalization and visualization of single-cell RNA-seq data in R. Bioinformatics. 2017;33(8):1179–86. doi: 10.1093/bioinformatics/btw777 28088763 PMC5408845

[30] Valero-Mora PM. ggplot2: Elegant Graphics for Data Analysis. J Stat Soft. 2010;35. doi: 10.18637/jss.v035.b01

[31] Gu Z. Complex heatmap visualization. iMeta. 2022;1(3):e43. doi: 10.1002/imt2.43 38868715 PMC10989952

[32] Hadley W, Thomas Lin P, Dana S. scales: Scale functions for visualization. 2023. Available from: https://github.com/r-lib/scales

[33] Love MI, Huber W, Anders S. Moderated estimation of fold change and dispersion for RNA-seq data with DESeq2. Genome Biol. 2014;15(12):550. doi: 10.1186/s13059-014-0550-8 25516281 PMC4302049

[34] Robinson MD, McCarthy DJ, Smyth GK. edgeR: a Bioconductor package for differential expression analysis of digital gene expression data. Bioinformatics. 2010;26(1):139–40. doi: 10.1093/bioinformatics/btp616 19910308 PMC2796818

[35] Paulson JN, Stine OC, Bravo HC, Pop M. Differential abundance analysis for microbial marker-gene surveys. Nat Methods. 2013;10(12):1200–2. doi: 10.1038/nmeth.2658 24076764 PMC4010126

[36] Ritchie ME, Phipson B, Wu D, Hu Y, Law CW, Shi W, et al. limma powers differential expression analyses for RNA-sequencing and microarray studies. Nucleic Acids Res. 2015;43(7):e47. doi: 10.1093/nar/gkv007 25605792 PMC4402510

[37] Segata N, Izard J, Waldron L, Gevers D, Miropolsky L, Garrett WS, et al. Metagenomic biomarker discovery and explanation. Genome Biol. 2011;12(6):R60. doi: 10.1186/gb-2011-12-6-r60 21702898 PMC3218848

[38] Nickols WA, Kuntz T, Shen J, Maharjan S, Mallick H, Franzosa EA, et al. MaAsLin 3: Refining and extending generalized multivariable linear models for meta-omic association discovery. bioRxiv. 2024. doi: 10.1101/2024.12.13.628459 39713460 PMC11661281

[39] Benjamini Y, Hochberg Y. Controlling the False Discovery Rate: A Practical and Powerful Approach to Multiple Testing. J R Stat Soc Series B: Stat Methodol. 1995;57(1):289–300. doi: 10.1111/j.2517-6161.1995.tb02031.x

