# Supplementary material for: MetaDAVis: An R shiny application for metagenomic data analysis and visualization
Source: PLoS One. 2025 Apr 7;20(4):e0319949. doi: 10.1371/journal.pone.0319949 (PMC11975103; doi:10.1371/journal.pone.0319949)
Supplement: S1 Table — (DOCX) [file pone.0319949.s001.docx]

**S1 Table. List of R packages used to develop MetaDAVis.**

| **R Packages** | **Used for** | **Web link** |
| --- | --- | --- |
| shiny | To develop the web and interactive application | <https://github.com/rstudio/shiny> |
| DT | Interface to the data tables | <https://github.com/rstudio/DT> |
| shinyFiles | A server-side file system viewer for shiny | <https://github.com/thomasp85/shinyFiles> |
| shinythemes | To use the shiny themes | <https://github.com/rstudio/shinythemes> |
| ggplot2 | To create plots and graphics | <https://github.com/tidyverse/ggplot2> |
| phyloseq | To explore microbiome profiles for alpha and beta diversity | <https://github.com/joey711/phyloseq> |
| ggpubr | Do the graphics for the correlation plot | <https://github.com/kassambara/ggpubr> |
| vegan | The beta diversity orientation methods | <https://github.com/vegandevs/vegan> |
| ggfortify | To plot PCA in 2D | <https://github.com/sinhrks/ggfortify> |
| plotly | To plot PCA in 3D | <https://github.com/plotly/plotly.R> |
| ggplotify | Convert plot to ggplot object | <https://github.com/GuangchuangYu/ggplotify> |
| reshape2 | To transform data into a different structure | <https://github.com/hadley/reshape> |
| tibble | To convert row names to column | <https://github.com/tidyverse/tibble> |
| scales | Scale functions visualization in a heatmap | <https://github.com/r-lib/scales> |
| dunn.test | Multiple comparisons using rank sums (used in the Kruskal-Wallis test) | <https://github.com/cran/dunn.test> |
| tidyr | Creating tidy data, where each column is a variable, each row is an observation | <https://github.com/tidyverse/tidyr> |
| dplyr | Data manipulation: adds new variables that are functions of existing variables | <https://github.com/tidyverse/dplyr> |
| devtools | To install several R packages | <https://github.com/r-lib/devtools> |
| patchwork | Adding multiple plots together | <https://github.com/thomasp85/patchwork> |
| RColorBrewer | To select the colors | <https://cran.r-project.org/web/packages/RColorBrewer/index.html> |
| zip | To extract the output to zip file | <https://cran.r-project.org/web/packages/zip/index.html> |
| GGally | Creating correlation plots | <https://github.com/ggobi/ggally> |
| BiocManager | To install Bioconductor packages | [https://bioconductor.org/packages/BiocVersion/](https://www.bioconductor.org/packages/BiocVersion/) |
| ComplexHeatmap | Creating heatmap | [https://bioconductor.org/packages/ComplexHeatmap/](https://www.bioconductor.org/packages/ComplexHeatmap/) |
| qvalue | Estimates for false discovery used in statistical analysis | <https://bioconductor.org/packages/qvalue/> |
| DESeq2 | Statistical analysis for two groups or sets | <https://bioconductor.org/packages/DESeq2/> |
| edgeR | Statistical analysis for two groups or sets | <https://bioconductor.org/packages/edgeR/> |
| limma | Statistical analysis for two groups or sets | <https://bioconductor.org/packages/limma/> |
| metagenomeSeq | Statistical analysis for two groups or sets | [https://bioconductor.org/packages/metagenomeSeq/](https://www.bioconductor.org/packages/metagenomeSeq/) |
| lefser | Statistical analysis for two groups or sets | <https://github.com/waldronlab/lefser> |
| maaslin3 | Statistical analysis for two groups or sets | <https://github.com/biobakery/biobakery/wiki/maaslin3> |
| bluster | Used in UMAP for creating k-means and graph-based clustering | [https://bioconductor.org/packages/bluster/](https://www.bioconductor.org/packages/bluster/) |
| mia | Used for data wrangling in t-SNE and UMAP | [https://bioconductor.org/packages/mia/](https://www.bioconductor.org/packages/mia/) |
| scater | Creating t-SNE and UMAP plots | [https://bioconductor.org/packages/scater/](https://www.bioconductor.org/packages/scater/) |
| microbiome | Utilities for microbiome analysis | <https://bioconductor.org/packages/microbiome/> |
| microbiomeutilities | Pairwise comparison using a non-parametric test (Wilcoxon test) in alpha diversity | <https://github.com/microbiome/microbiome/> |
