# Supplementary material for: MetaDAVis: An R shiny application for metagenomic data analysis and visualization
Source: PLoS One. 2025 Apr 7;20(4):e0319949. doi: 10.1371/journal.pone.0319949 (PMC11975103; doi:10.1371/journal.pone.0319949)
Supplement: S2 Table — (DOCX) [file pone.0319949.s002.docx]

**S2 Table. Output table for the significant taxa by using various methods.**

|  | **Wilcoxon Rank Sum test** | **t-test** | **metagenomeSeq** | **DESeq2** | **Limma-Voom** | **edgeR** | **LefSe** | **MaAsLin3** |
| --- | --- | --- | --- | --- | --- | --- | --- | --- |
| OUT (Taxa) | **✓** | **✓** | **✓** | **✓** | **✓** | **✓** |  |  |
| Present_in_no_of_CD (Condition1) | **✓** | **✓** | **✓** |  |  |  |  |  |
| Present_in_no_of_HC (Condition2) | **✓** | **✓** | **✓** |  |  |  |  |  |
| Counts_in_HC |  |  | **✓** |  |  |  |  |  |
| Counts_in_CD |  |  | **✓** |  |  |  |  |  |
| Mean /Mean_relative_frequency_CD (Condition1) | **✓** | **✓** |  |  | **✓** |  |  |  |
| Mean / Mean_relative_frequency_HC (Condition2) | **✓** | **✓** |  |  | **✓** |  |  |  |
| All_mean / All_mean_relative_frequency | **✓** | **✓** | **✓** | **✓** | **✓** |  |  |  |
| Difference_between_means | **✓** | **✓** |  |  |  |  |  |  |
| fold_change | **✓** | **✓** | **✓** | **✓** | **✓** | **✓** |  |  |
| log2FC | **✓** | **✓** | **✓** | **✓** | **✓** | **✓** |  |  |
| PValue | **✓** | **✓** | **✓** | **✓** | **✓** | **✓** |  | **✓** |
| FDR or q_value | **✓** | **✓** | **✓** | **✓** | **✓** | **✓** |  | **✓** |
| Scores |  |  |  |  |  |  | **✓** |  |
