# Supplementary figures and images for: MetaDAVis: An R shiny application for metagenomic data analysis and visualization
Source: PLoS One. 2025 Apr 7;20(4):e0319949. doi: 10.1371/journal.pone.0319949 (PMC11975103; doi:10.1371/journal.pone.0319949)

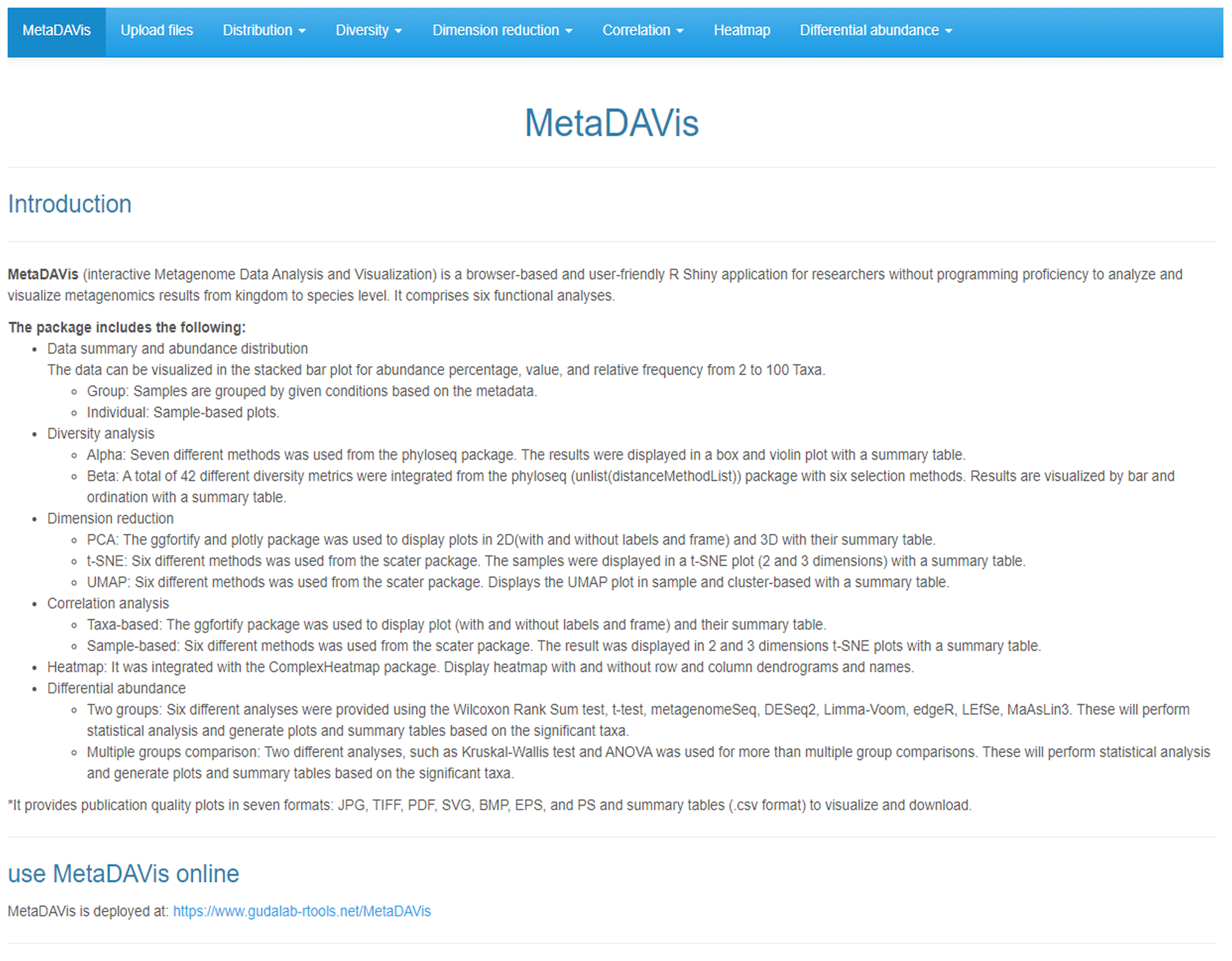

Supplement: S1 Fig — (TIF) [file pone.0319949.s003.tif]

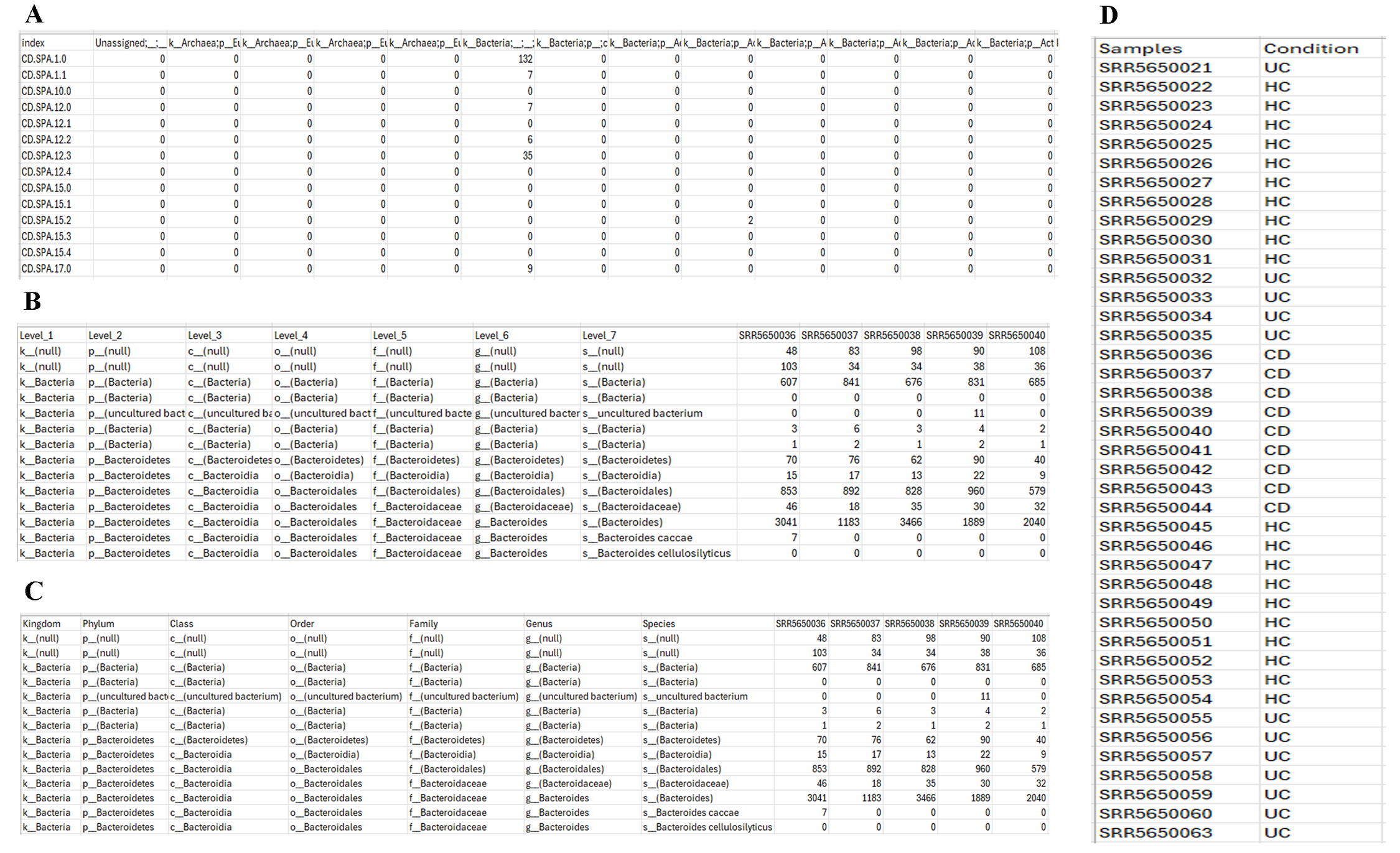

Supplement: S2 Fig — (A) Qiime 2 output (Level 7), (B) MEGAN output file, (C) user-defined file format, and (D) metadata file applicable to all three formats. (TIF) [file pone.0319949.s004.tif]

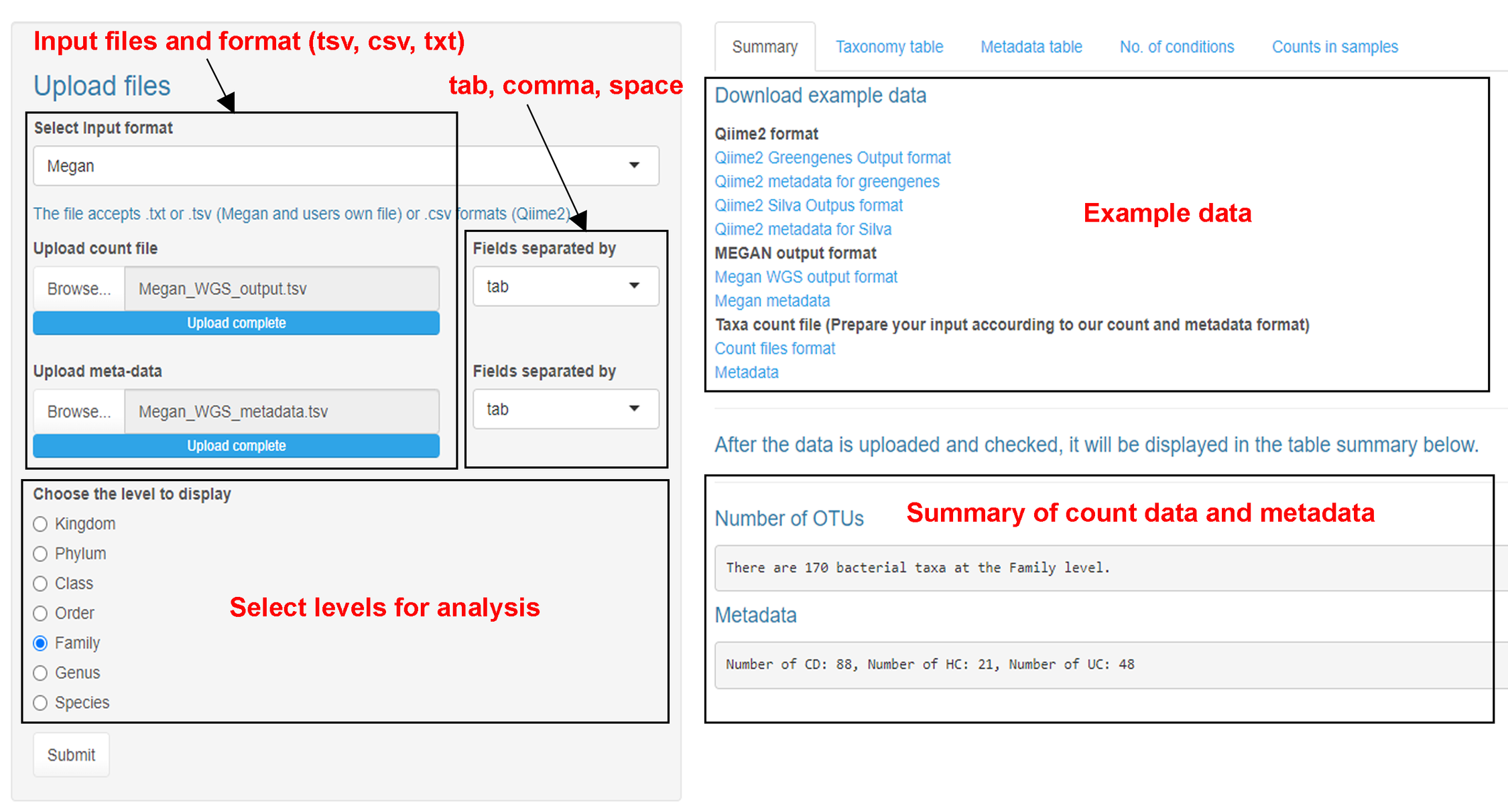

Supplement: S3 Fig — Example data were provided for Qiime2, MEGAN output format. If users have a different output format, they should be prepared according to the taxa count file format. (TIF) [file pone.0319949.s005.tif]

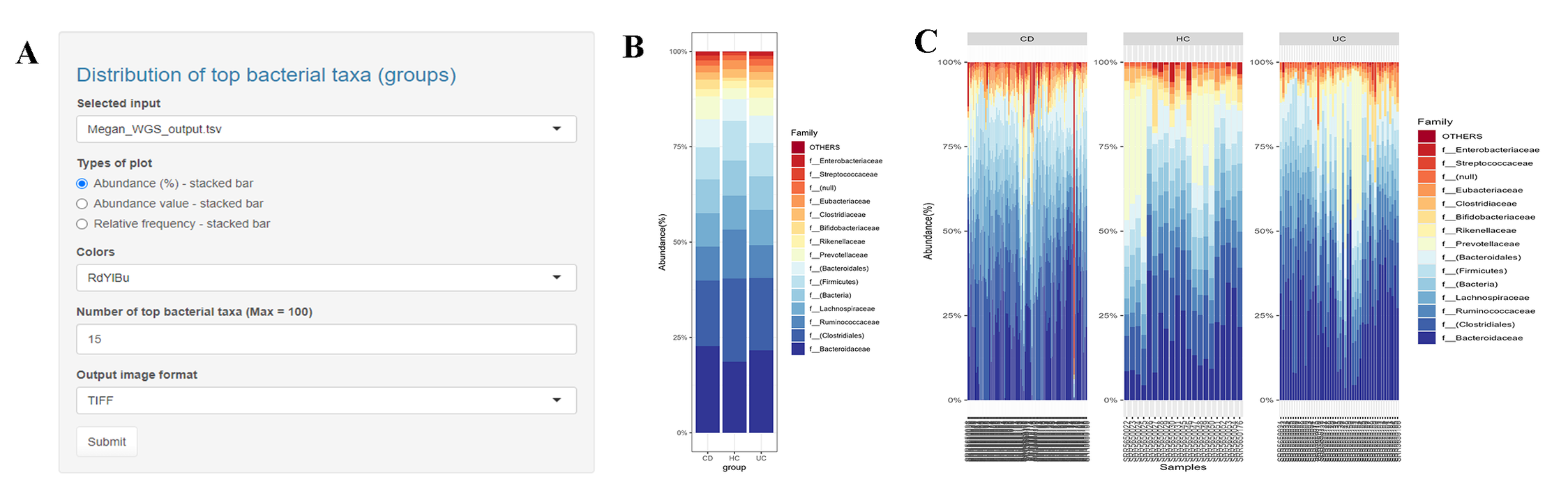

Supplement: S4 Fig — (A) Choice of distribution plot and output format; (B) Box plot for comparison groups; and (C) Box plots for individual samples. (TIF) [file pone.0319949.s006.tif]

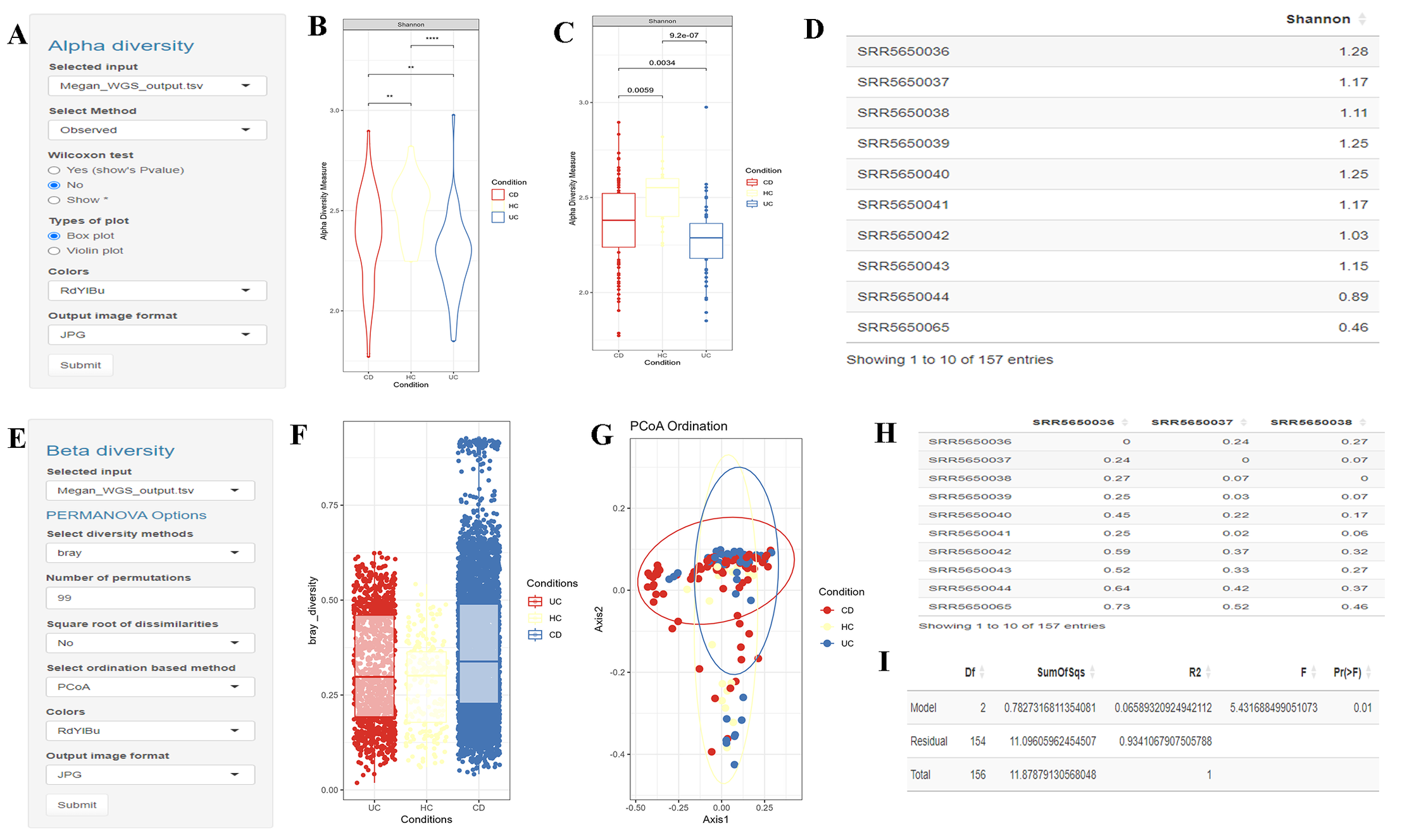

Supplement: S5 Fig — (A) Choice of alpha diversity method from seven different methods such as Observed, Chao1, ACE, Shannon, Simpson, Inverse Simpson, Fisher or All_combined; (B) Violin plot showing the Simpson diversity; (C and D) Shannon diversity plot with corresponding values in a table; (E) Selected choice of beta diversity methods (bray-curtis) with other options;; corresponding (F) bar plot (G) dot plot (H) values in a table and (I) adonis2 function table. (TIF) [file pone.0319949.s007.tif]

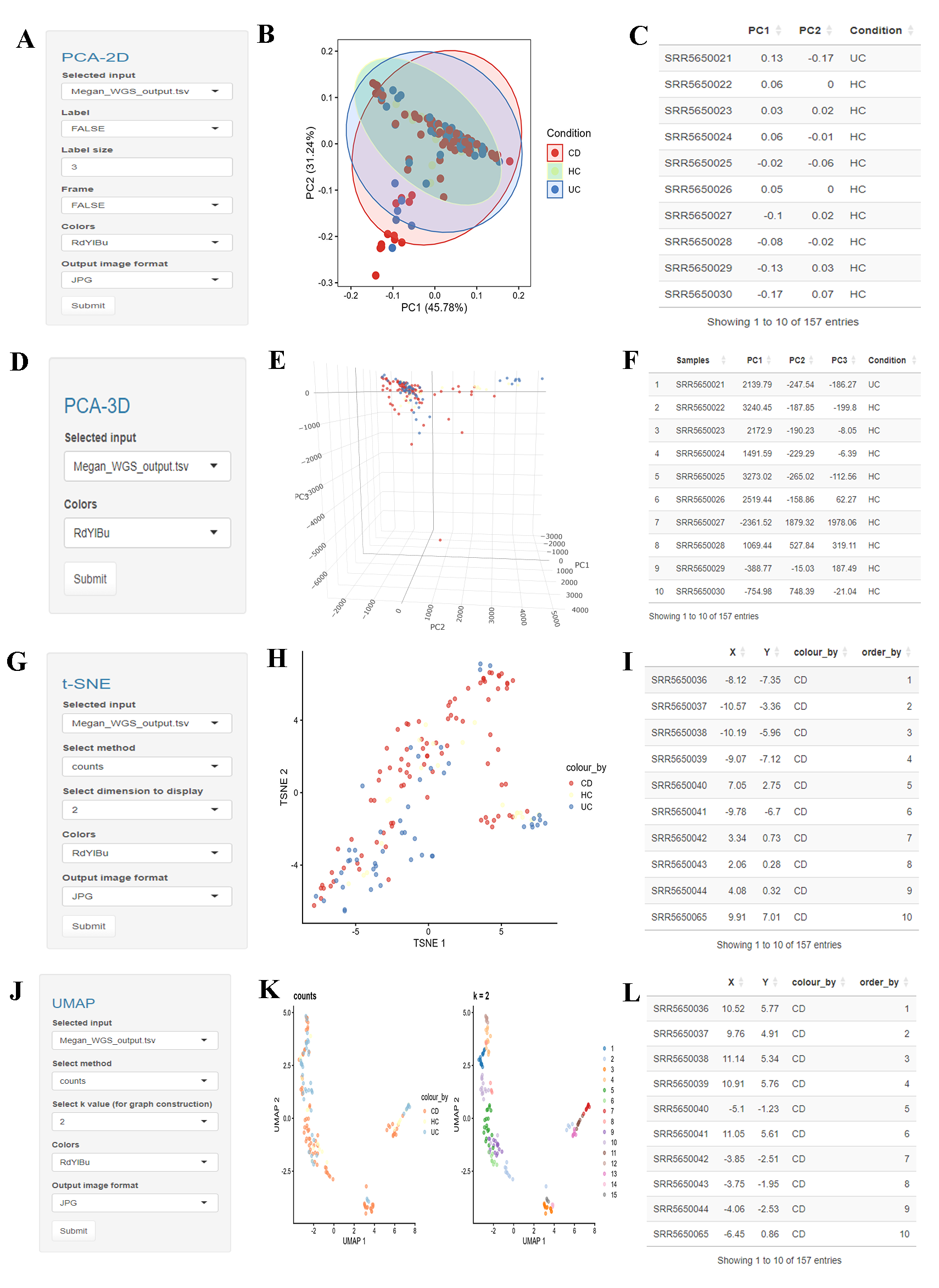

Supplement: S6 Fig — (A-C) The choice of PCA-2D and the plot with frames and summary table of sample coordinate positions shown for PC1 and PC2; (D-F) PCA 3-D selection and the 3-D plot and summary table of sample coordinate positions shown for PC1, PC2, and PC3; (G-I) t-SNE with selected options, two-dimension plots with the selected rcl method, and corresponding summary table; (J-L) UMAP with selected options, condition-based and cluster-based (K = 5) UMAP plots with selected rcl method, and corresponding summary table. (TIF) [file pone.0319949.s008.tif]

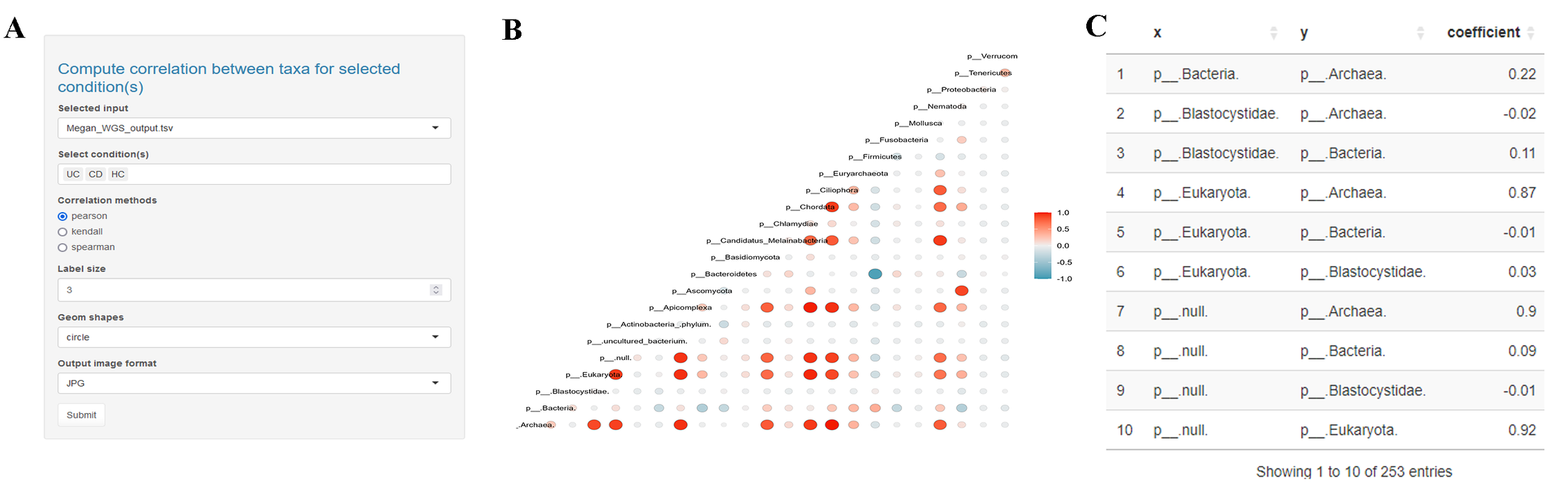

Supplement: S7 Fig — (A) Input selection for taxa-based correlation analysis using the condition option; (B) Taxa-based correlation plot using Pearson method; and (C) summary table. A similar type of method selection and results were implemented in sample-based correlation analysis. (TIF) [file pone.0319949.s009.tif]

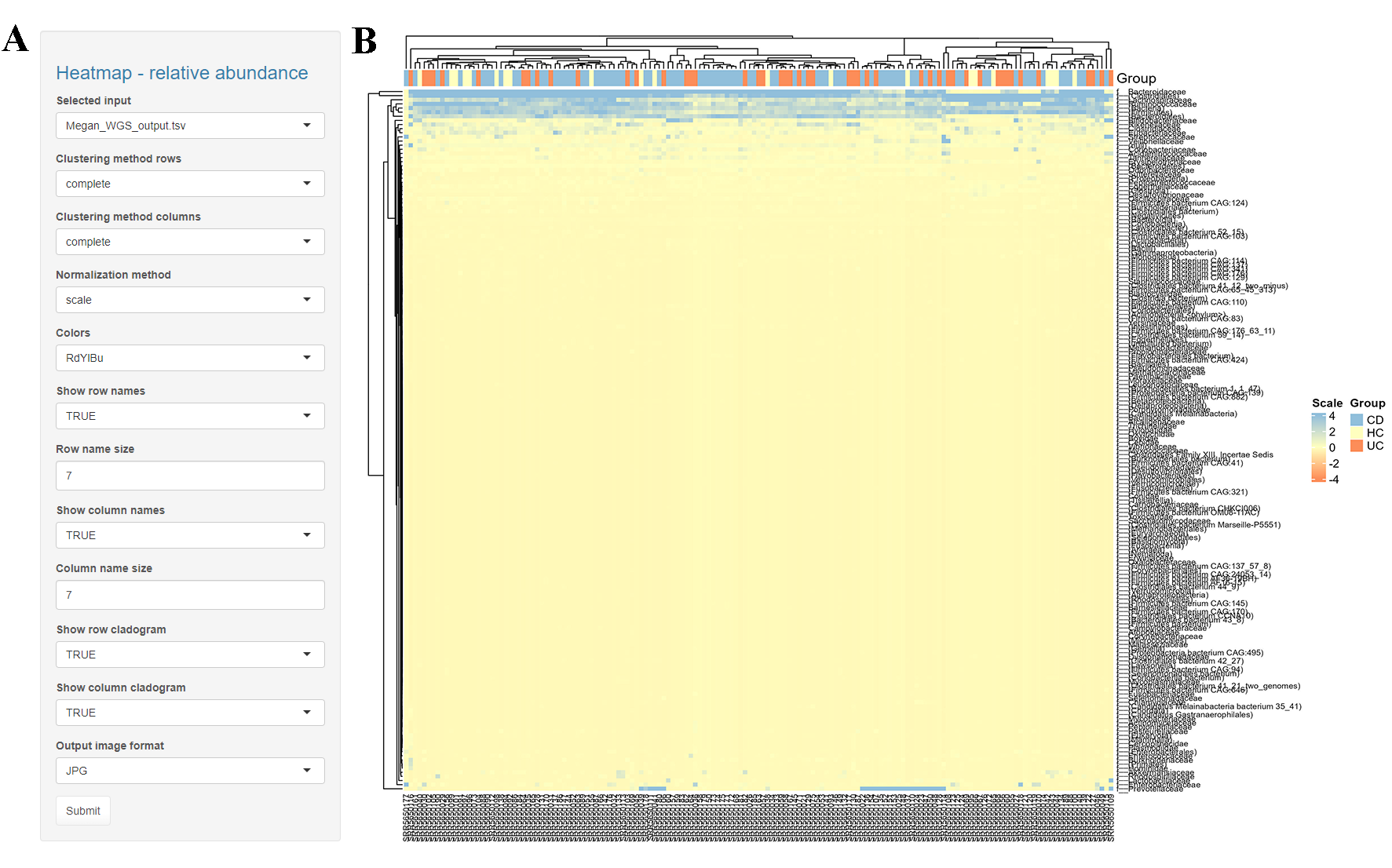

Supplement: S8 Fig — (A) Input selection for heatmap analysis, user can adjust the row and column text size and cladograms; and (B) Heatmap for the selected taxonomy level shows sample names in rows and family names in columns with a cladogram. Scale values represent colors in the heatmap and condition groups. (TIF) [file pone.0319949.s010.tif]

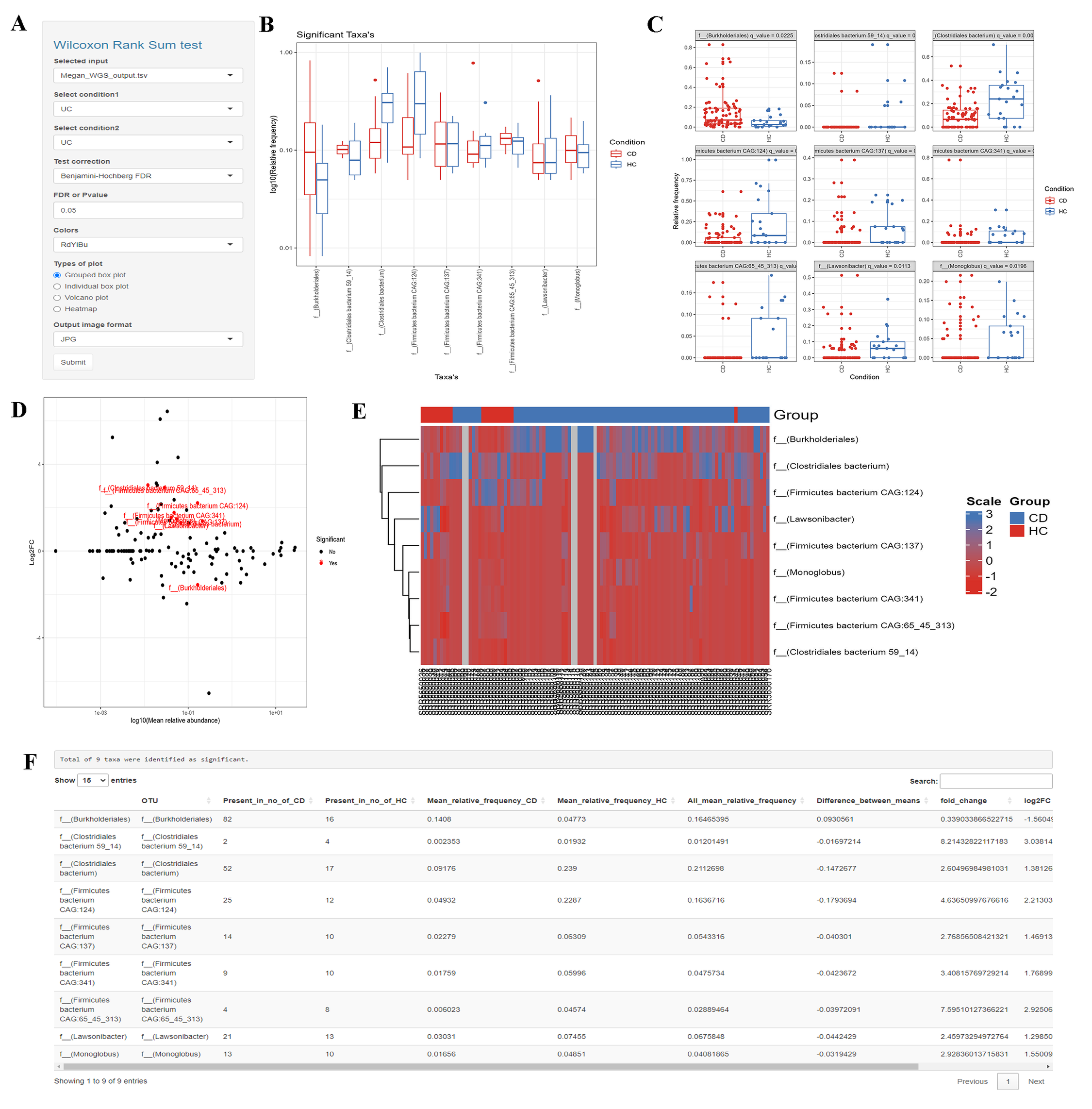

Supplement: S9 Fig — (A) Input selection of Wilcoxon Rank Sum test; (B) Grouped box plot, x-axis represents taxa and y-axis represents log10(relative frequency); (C) An individual box plot for each taxon, x-axis represents the condition and y-axis represents relative frequency; (D) Volcano plot, x-axis represents log10(mean relative abundance) and y-axis represents Log2FC; (E) Heatmap for significantly identified taxa; (F) Summary table for the Wilcoxon Rank Sum test. Similar input is needed for the remaining pairwise methods such as, metagenomeSeq, DESeq2, Limma-Voom and edgeR and multiple group comparison Kruskal-Wallis test and ANOVA. (TIF) [file pone.0319949.s011.tif]
